# Efficient design of energy microgrid management system: A promoted Remora optimization algorithm-based approach

**DOI:** 10.1016/j.heliyon.2023.e23394

**Published:** 2023-12-10

**Authors:** Hua Zhang, Yingying Ma, Keke Yuan, Majid Khayatnezhad, Noradin Ghadimi

**Affiliations:** a School of Computer Science and Engineering, Hunan University of Information Technology, ChangSha, 410151, China; b School of Electronic Science and Engineering, Hunan University of Information Technology, ChangSha, 410151, China; c Young Researchers and Elite Club, Ardabil Branch, Islamic Azad University, Ardabil, Iran

**Keywords:** Optimal energy management strategy, Microgrids, Promoted remora optimization algorithm, Fuel cell, Battery storage, Photovoltaic system

## Abstract

Microgrids are a promising solution for decentralized energy generation and distribution, offering reliability, efficiency, and resilience. These small-scale power systems can operate independently or connect to the main grid, providing greater reliability and resilience. However, integrating renewable energy into microgrids presents challenges due to their unpredictable nature and fluctuating load of electricity. Energy management strategies play a crucial role in optimizing the operation of microgrids, aiming to balance electricity supply and demand, maximize renewable energy utilization, and minimize operational costs. Various approaches have been proposed for energy management in microgrids, including optimization algorithms, machine learning techniques, and intelligent control systems. This study proposes an optimized and efficient strategy for microgrids operating in both independent and grid-connected modes, focusing on microgrids that utilize a combination of solar and green energy sources. The proposed approach, based on the Promoted Remora Optimization (PRO) algorithm, aims to meet load power requirements at the lowest possible cost while ensuring constant DC bus voltage and safeguarding batteries against overcharging and depletion. The CRO method effectively optimized the charging process, maintaining a consistent level of charge and achieving a final SoC of 33.37 %–33.60 %. It also demonstrated high system efficiency, with an average of 87.99 %, and a range of 87.80 %–88.03 %. The optimizer efficiency ranged from 83.12 % to 86.52 %, with an average of 86.46 %. The CRO method also achieved reasonable operating costs, with a cost per power of $0.1687/kW to $0.1699/kW and a daily cost of $1,379,595 to $1,479,998. Overall, the CRO method showed promise in optimizing the charging process in terms of efficiency and cost-effectiveness. Comparative analysis with existing literature is conducted to evaluate the effectiveness of the proposed approach, demonstrating its superior results compared to other energy management strategies for microgrids. This study contributes to the field of microgrid energy management by providing a novel approach based on the PRO algorithm and demonstrating its effectiveness through comparative analysis.

## 1 Introduction

With the rising demand for electricity and mounting apprehensions regarding climate change and environmental sustainability, there is a growing emphasis on the advancement of decentralized energy generation and distribution systems [1]. Microgrids have become a viable and promising solution for delivering dependable, resilient, and efficient electricity to buildings, communities, and industrial establishments [2]. Microgrids refer to power systems of a smaller scale that can function in two modes: linked to the primary grid or autonomously in the mode of island [3]. Frequently, a combination of renewable energy sources, including solar photovoltaics (PV), wind turbines, and biomass, is integrated, alongside other clean energy technologies such as energy storage systems and demand response capabilities [4].

The incorporation of energy sources that are renewable into microgrids presents both advantages and disadvantages. The generation of energy that is renewable has been frequently branded by intermittency and variability, primarily attributable to factors such as meteorological conditions and diurnal cycles [5]. Moreover, the fluctuation of electricity load poses a significant challenge in effectively aligning supply and demand. To tackle these challenges, it is imperative to implement efficient energy management strategies that can effectively optimize the functioning of microgrids [6].

The main goal of energy management strategies is achieving equilibrium between the electricity supply and demand within the microgrid, while simultaneously optimizing the utilization of renewable energy sources, minimizing operational expenses, and guaranteeing consistent and dependable performance [7]. Different methods are suggested for management of energy in microgrids [8]. These include optimization algorithms [9], machine learning techniques [10], and intelligent control systems [11]. These strategies strive to adaptively manage energy resources, forecast renewable energy generation, optimize power dispatch, and provide robust solutions under varying conditions [12].

Microgrids, composed of various energy sources like fuel cells, battery storage, and photovoltaic systems, offer a promising solution for sustainable and reliable energy supply [13]. But, the intermittent notion of renewable sources of energy and fluctuating load of electricity pose operational and economic challenges for microgrid systems [14]. Therefore, optimizing the energy management strategy becomes crucial to ensure cost-effectiveness, stability, and overall system performance [15].

Several investigations are carried out on the topic of Energy Microgrid Management Systems, focusing on various aspects of microgrid operation and optimization [16]. Researchers have proposed different strategies and techniques to effectively manage the energy flow, balance supply, and demand, and maximize the utilization of renewable energy sources within microgrids [17].

In this context, Abd-Elhaleem et al. [18] proposed an enhanced management technique of power for PHEVs (Plug-in Hybrid Electric Vehicles) that is composed of a long-standing management method of power and a temporary controller that is intelligent. The long-standing management of power used a CEGPSO (Chaotic Enhanced Generalized Particle Swarm Optimization) approach for optimizing the diesel and torques of motor engine [19]. A system of control that was based on five mode rules was used to reduce computation time that the CIGPSO estimated the optimized values of torques in the engine and motor in a mode of hybrid. The temporary controller was made utilizing an IT2TSK fuzzy (interval type-2 Takagi-Sugeno-Kang) technique that is dependent on experts of human to vanquish doubts of varied driving circumstances. The suggested technique reduced utility of energy in comparison with several methods like enhanced multi-objective Particle Swarm Optimizers and generalized Particle Swarm Optimizer [17]. However, the limitations of this work included the fact that the proposed strategy had only been tested through simulations utilizing actual data of the battery, motor, and engine, and had not been tested in real-world driving conditions.

Huang et al. [20] proposed a two-stage energy management method for a heat-electricity integrated energy system (HE-IES) considering dynamic pricing of the master-slave game and operation strategy optimization. The method aimed to enhance the efficacy of energy and procedure exoense of the model. The first stage established an overall broad efficacy of energy regarding the exergy belongings of electric and thermal energy, and the second stage included a multi-objective optimizer of day-ahead preparation and master-slave game dynamic valuing system of actual-time management of energy according to the interaction optimizer of source load for energy of hybrid. The proposed approach could improve utility of energy that is renewable, decrease the expense of both users and ESP and encourage the interaction that is benign between load in IES and equipment of energy that has the potential to enhance the energy efficacy of system operation. On the other hand, it should be noted that the efficiency of the proposed method might depend on various factors such as the specific characteristics of the HE-IES, the availability and reliability of data, and the accuracy of the models used.

Aguila-Leon [21] presented a method for incorporating optimal artificial networks into a self-adjustable management system of energy for improving microgrid efficiency. The suggested system consisted of a series of ANNs systematized into a configuration of cascade and a PSO algorithm optimized for every ANN. The system aimed to provide and assess information to the management system of the energy. The system was applied to environment of MATLAB/Simulink and fed with investigational data. The results showed that the suggested model decreased errors by 56 % and 59 % for multiple-step and single-step forecast of energy variable estimators. However, the limitations of this work could be that the proposed model was tested only with experimental data, and it might not apply to other scenarios. Additionally, the proposed model might require a high computational cost, which might not be feasible for real-time applications.

Merabet et al. [22] proposed an improved energy management system for a wind energy and hybrid solar microgrid with an option of grid-connected and storage of battery. The system used a variable named the issue of contribution to provide an enough amount of necessary power from the battery on the basis of the main grid's electricity expense. A shifting method of load was combined into the management of energy for improving the cost of energy. The structure accurately computed the energy cost using the efficiencies of the elements. Simulations showed that the proposed management system of energy was superior to the traditional one in decreasing battery degradation cost, cost of energy, and increasing battery lifespan. However, it is important to note that the effectiveness of the proposed energy management system may depend on various factors such as the size of the microgrid, the availability and reliability of renewable energy sources, and the electricity price of the main grid. These factors may affect the performance of the system and should be taken into consideration when implementing the proposed method.

Seydali et al. [23] proposed an optimal energy management strategy (EMS) for a DC microgrid that combined multiple power sources, like renewables, fuel cells, and storage systems of battery, was proposed. The EMS was based on the Salp swarm algorithm (SSA), which offered advantages including convergence properties and reduced computing complexity. The paper provided a detailed step-by-step design of the proposed method and conducted hardware-in-the-loop (HIL) tests to validate its performance. A comparison was made between the proposed EMS, the state machine control strategy (SMC), and EMS based on particle swarm optimization (PSO) in terms of system efficiency, fuel consumption, and power quality. The results obtained demonstrated the superiority of the proposed EMS in terms of fuel saving and power quality. However, it should be noted that further testing and validation in different scenarios are required, and there may be challenges in implementing the proposed EMS in real-world applications.

Although several energy management strategies have been proposed for microgrids, there is a need for further improvement in terms of efficiency and cost-effectiveness. Existing methods often fail to adequately address the unpredictability of renewable energies, protect batteries from overcharging and depletion, and optimize the overall microgrid system effectiveness. To bridge this research gap, our study introduces a novel approach based on a modified version of a metaheuristic algorithm. The motivation behind this research is to develop an optimal energy management strategy that overcomes the limitations of existing methods and maximizes the performance of microgrids.

The main objective of this study is to propose an optimal and efficient energy management strategy (EMS) for a combined solar and green energy microgrid. The study aims to address the instability and economic issues associated with renewable energies and load electricity in microgrids. The main contributions of this study are as follows.

− Promoted Remora Optimization Algorithm: The study introduces a novel metaheuristic algorithm called the Promoted Remora Optimization (PRO) algorithm, which has been modified and improved specifically for the energy management of microgrids. This algorithm is designed to optimize power supply, minimize costs, maintain a constant DC bus voltage, protect the battery storage from overcharging and depletion, and enhance the overall system's effectiveness.
− Cost-Effective Performance: The proposed EMS aims to efficiently manage the power requirements of the load at the lowest possible cost while ensuring stable and reliable operation of the microgrid. By optimizing the use of solar and green energy sources, as well as the battery storage and fuel cell system, the study focuses on achieving a cost-effective performance of the microgrid.
− Simulation and Comparison: The suggested method is evaluated through simulation based on a one-day planning perspective. The study compares the simulation results of the proposed Promoted Remora Optimization Algorithm with other existing methods studied in the literature. This allows for an assessment of the effectiveness and superiority of the proposed EMS in terms of cost optimization and system performance.

In general, the main objective of this study is to propose an optimal and efficient EMS for a combined solar and green energy microgrid. The main contributions include the introduction of the Promoted Remora Optimization Algorithm, the focus on cost-effective performance, and the evaluation and comparison of the proposed method with existing approaches.

## 2 Model of the system

**Fig. 1:**
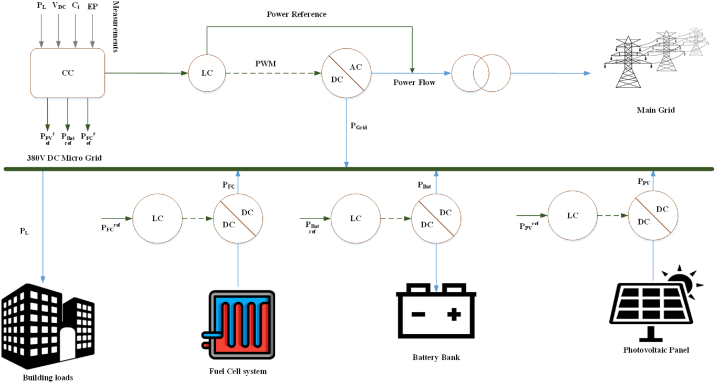
Complete block diagram of the analyzed system.

The low-voltage Energy Microgrid System exhibits considerable potential as a feasible solution for providing electricity to buildings in an environmentally sustainable and economically efficient manner. The system exhibits significant potential as a feasible solution. To ensure a consistent and dependable power supply, the system integrates the power generation functionalities of a Photovoltaic (PV) system, a Fuel Cell (FC), and a Battery Energy Storage System (BESS). Moreover, the system possesses the capacity to engage in power exchange with the primary grid, thereby enabling the optimization of renewable energy utilization and consequent reduction in electricity expenditure. Fig. (1) shows a complete block diagram of the analyzed system.

### 2.1 Photovoltaic system

The photovoltaic (PV) system holds significant importance as a fundamental element within the low-voltage Energy Microgrid System. The photovoltaic (PV) system utilizes solar energy and transforms it into electrical energy through the utilization of photovoltaic cells. When subjected to sunlight, these cells produce direct current (DC) electricity. The photovoltaic (PV) system is regulated through the utilization of a boost converter, which effectively adjusts the voltage and current levels to align with the specific demands of the microgrid system. The photovoltaic (PV) system is widely regarded as a dependable and highly efficient method for harnessing electricity from sustainable and renewable energy sources. In this scenario, a 12-pulse inverter is utilized to mitigate voltage total harmonic distortion (THD) when supplying power to a three-phase alternating current (AC) load [24].

To attain the desired output power of a Photovoltaic (PV) system within the proposed energy management system, it is imperative to take into account various factors. The power generated by a photovoltaic (PV) system is contingent upon a multitude of factors, including temperature, solar irradiance, and the specific characteristics of the system itself. The single-diode equivalent circuit model is widely employed as the predominant method for estimating the power output of photovoltaic (PV) systems. The present model is grounded on the fundamental physical principles governing the operation of a photovoltaic (PV) cell and can be mathematically represented as follows:

(1) $$P_{PV}=V_{PV}\times I_{PV}$$

where, the variables $V_{pv}$ describes the output voltage, and $I_{pv}$ represent the output current of the photovoltaic (PV) system and is achieved as follows:

(2) $$I_{PV}=I_{L}-I_{0}\times \left[\exp\left(\frac{\left(V+I\times R_{s}\right)}{n\times V_{t}}\right)-1\right]-\left(V+I\times R_{s}\right)/R_{sh}$$

where, the photocurrent, denoted as $I_{L}$, represents the electrical current produced by the incident solar irradiance, the symbol $I_{0}$ represents the reverse saturation current of the diode, the voltage across the terminals of the photovoltaic (PV) cell is denoted as V, the variable $R_{s}$ represents the resistance in a series circuit, the symbol n represents the ideality factor of a diode, and the thermal voltage, denoted as $V_{t}$, can be expressed as k×T/q, where k represents Boltzmann's constant, T denotes the temperature measured in Kelvin, and q signifies the elementary charge, and the variable $R_{sh}$ represents the shunt resistance.

To acquire accurate values for the input parameters ($I_{L}$, $I_{0}$, n, $R_{s}$, $R_{sh}$), one may consult the manufacturer's datasheet or conduct experimental characterization of the photovoltaic (PV) system. The present study employs the characteristics outlined in Table 1 [25] for the photovoltaic system under investigation [26]. Table 1 indicates the outlined characteristics of the studied PV system

**Table 1:** Outlined characteristics of the studied PV system.

Parameter	Feasible Value
$I_{L}$	6.5 A
$I_{0}$	1e-9 A
n	1.3
$R_{s}$	0.05 Ω
$R_{sh}$	200 Ω

To provide the appropriate output voltage, the photovoltaic system is connected to a boost converter, as was previously explained.

### 2.2 Fuel cell (FC) system

A photovoltaic (PV) system is designed to harness solar energy and convert it into electrical power through the utilization of photovoltaic cells. When subjected to illumination, these cells generate electrical energy through a process referred to as direct current (DC) generation. The utilization of a boost converter is employed in the operation of the photovoltaic (PV) system. This converter is designed to optimize the levels of voltage and current to meet the specific requirements of the microgrid system.

(3) $$P_{FC}=S\times n\times V_{FC}\times I$$

where, n describes the single cells' quantity, S represents the available area, I is the fuel cell current and based on Butlere-Volmer and [27], can be achieved as follows [25]:

(4) $$I={2\times I}_{0}\times \sinh\left(\frac{n_{e}\times F\times V_{act,cell}}{2\times R\times T}\right)$$

Here, $I_{0}$ represents the exchange current density, and $n_{e}$ specifies the moved moles of electrons.

At last, the attainment of the output voltage of the Solid Oxide Fuel Cell (SOFC) has been accomplished through the methods outlined based on [28] as follows:

(5) $$V_{FC}=E_{0}-A\;\sinh^{-1}\left(\frac{I}{{2\times I}_{0}^{c}}\right)-A\times \sinh^{-1}\left(\frac{I}{{2\times I}_{0}^{a}}\right)-I\times R_{\Omega }+B\times \ln\left(\frac{I_{L}-L}{I_{L}}\right)$$

where, A represents the slope of the Tafel line, $I_{L}$ determines the current density restriction, B determines a factor related to the working state of the fuel cell, $R_{\Omega }$ signifies the area resistance of the fuel cell, and $I_{0}^{c}$ and $I_{0}^{a}$ represent the current density exchange of the cathode and anode, respectively [29]. Table 2. Indicates the outlined characteristics of the studied FC system

**Table 2:** Outlined characteristics of the studied FC system.

Parameter	Feasible Value
$E_{0}$	0.7 V
A	0.05 V
B	0.1 V
$I_{0}^{a}$	0.02 $A/cm^{2}$
$I_{0}^{c}$	0.03 $A/cm^{2}$
$R_{\Omega }$	0.5 $k\Omega \;cm^{2}$
$I_{L}$	0.1 $A/cm^{2}$

### 2.3 Lithium-ion battery energy storage system (BESS)

The Battery Energy Storage System (BESS) is responsible for the storage of surplus electricity produced by the Fuel Cell (FC) and Photovoltaic (PV) system, allowing for its utilization at a later time. The technology employs lithium-ion batteries, which possess notable attributes such as high energy density and efficiency. The Battery Energy Storage System (BESS) has vital part in enhancing the stability of the microgrid system by offering supplementary power during instances of reduced solar energy generation or heightened electricity consumption. To regulate the power flow to and from the batteries, a buck/boost converter is utilized. The buck/boost converter is responsible for regulating the voltage and current levels to facilitate the efficient charging and discharging processes of the batteries. The output power of a BESS can be determined by employing the following equation:

(6) $$P_{BESS}=N_{bat}\times V_{BESS}\times I_{discharge}^{\max}$$

where, $N_{bat}$ specifies the total number of batteries connected to the system, $I_{discharge}^{\max}$ specifies the maximum current that each battery can deliver during discharge, and $V_{BESS}$ refers to the nominal voltage of each battery and is achieved as follows:

(7) $$V_{B}=E_{BESS}\pm R_{Bat}\times I_{BESS}$$

where, $E_{B}$ describes the battery potential, $I_{BESS}$ describes the current for the BESS, and $R_{B}$ specifies the real part of the resistance $Z_{B}$, and can be achieved as follows:

(8) $$R_{B}=\left|Z_{Bat}\right|\times \cos\;\rho_{Bat}$$

where, $\rho_{Bat}$ specifies the phase shift.

### 2.4 System output

The DC bus accessible energy ($E_{B}$) can be described as a function of the transferred power (P) using the formula:

(9) $${\dot{E}}_{B}=\sum P=P_{G}+P_{BESS}+P_{FC}+P_{PV}-P_{L}$$

where, $P_{BESS}$, $P_{G}$, $P_{FC}$ , $P_{L}$, and $P_{PV}$ represent the generated power by the grid, BESS, grid, fuel cell, load, and photovoltaic system, respectively.

## 3 Fitness function

### 3.1 Definition

The required power exceeded the generated Photovoltaic power, indicating that the power deficit was supplemented by the grid power, the fuel cell, and the BESS, following the selected energy management strategy. In terms of financial benefits, the micro-grid transitioned to the grid mode of operation, with the majority of the load being supplied by grid power. This occurred when either the load power exceeded the capacity of the microgrid's power resources or when the operational expenses associated with utilizing the power resources were significantly higher. The problem at hand can be framed as an optimization problem, wherein the objective is to minimize the costs associated with the operations. The optimization problem can be mathematically defined as follows [30]:

(10) $$Cost=\sum_{t=1}^{T}\left(C_{FC}(P_{FC}\left(t\right)+C_{Bat}\left(P_{Bat}\left(t\right)\right)+P_{Grid}\left(t\right)\times EP\left(t\right)\Delta T\right)$$

In the above scenario, the variable EP(t) denotes the pricing of electricity in the market at time t. ΔT represents the sample time. T represents the total number of power sources. N represents the entirety of time intervals, and $C_{FC}$, $C_{BESS}$, and $C_{i}$ represent the cost associated with the $i^{th}$ fuel cell (FC), battery energy storage system, and distributed generation (DG), respectively. The function encountered several limitations, such as constraints on power generation capacity and power equilibrium.

When the losses of the microgrid are disregarded, it is necessary for the total power generated by the power resources to be equal to the power consumed by the load at each time interval, t. Consequently, the limitation on power balance is outlined as follows:

(11) $$P_{L}=P_{PV}+P_{BESS}+P_{FC}+P_{G}$$

The utilization of microgrids is employed to share BESS, according to the rules of the energy management strategy. However, some studies promoting an economic energy management system (EMS) failed to incorporate SoC of the battery (state of charge) within the EMS. To mitigate the potential negative effects of excessive battery drain or overcharging, the Energy Management System (EMS) must integrate the state of charge as a crucial component within the fitness function. In this study, the assessment of the battery's state of charge was conducted utilizing the subsequent fitness function:

(12) $$F_{Cost}=\min\left(\sum_{t=1}^{T}\left(C_{FC}(P_{FC}\left(t\right)+C_{Bat}.P_{Bat}\left(t\right){\left(SoC\left(t\right)-SoC{\left(t\right)}_{opt}\right)}^{2}+P_{Grid}\left(t\right)\times EP\left(t\right)\Delta T\right)\right)$$

The variable $SoC{\left(t\right)}_{opt}$ represents the optimal value of the state of charge.

### 3.2 Constraints

Generation units possess distinct physical manufacturing capabilities that dictate their power supply capacity. The manufacturing capability of these units establishes a defined operational range in which they are capable of functioning and delivering power. The units must ensure that the power supplied to them remains within the specified range to uphold their optimal performance. The capacity of power generation units to deliver electricity is influenced by a multitude of factors, encompassing the design specifications and technical constraints inherent to these units. The factors encompassing the generator's performance include its capacity, efficiency, and operational limitations, such as temperature thresholds and mechanical stress thresholds.

Furthermore, the range of power that can be consistently generated is also influenced by the accessibility and caliber of fuel or energy sources. To optimize the performance and durability of the generation units, it is imperative to operate them within the designated power range. Utilizing the units below the lower threshold may give rise to suboptimal utilization and ineffective performance, thereby potentially engendering operational challenges. Conversely, exceeding the prescribed upper threshold of the power range may impose excessive stress on the units, thereby leading to potential equipment damage or failure. By adhering to the prescribed power range, generation units can optimize their performance, ensure operational reliability, and prolong their lifespan. The successful operation of the units necessitates meticulous monitoring, control, and coordination to guarantee that the power output remains within the predetermined range. This entails taking into account various factors, including fluctuations in demand, grid stability, and system requirements. Here,

(13) $$P_{G}\in \left[P_{G}^{\min},P_{G}^{\max}\right]$$

The operational expense of the generators is considered to follow an equation with a single variable of degree 2, as represented by Eq. (13).

(14) $$C_{i}={\left(a_{i}P_{Gi}\right)}^{2}+b_{i}P_{Gi}+c_{i}$$

where, $a_{i}$, $b_{i}$, and $c_{i}$ represent the cost coefficients.

As a result, the fitness function imposes restrictions on the power producers, specifically limiting the output of the fuel cell and the BESS. These constraints can be summarized as follows:

(15) $$P_{Bat}\in \left[P_{Bat}^{\min},P_{Bat}^{\max}\right]$$

(16) $$P_{FC}\in \left[P_{FC}^{\min},P_{FC}^{\max}\right]$$

(17) $$P_{G}=P_{L}-P_{Bat}-P_{FC}$$

The optimization variables considered in this study were the power reference of the generators, which included the power reference of the core grid. Furthermore, the inclusion of the main grid power reference was not considered an optimization parameter to ensure power equilibrium. The current investigation introduces a reformed model of the CRO for the intended purpose.

The photovoltaic system, the main grid power, and the fuel cell collectively fulfill a significant portion of the energy requirements. The primary function of the battery is to ensure a constant DC voltage for the bus by providing intermittent support. The utilization of flatness control methodology can be employed to achieve voltage stabilization, provided that the subsequent equation is utilized as the reference for battery power.

(18) $$P_{Bat}^{ref}={\dot{E}}_{B}^{req}+P_{L}-\left(P_{PV}+P_{G}+P_{FC}\right)$$

The variable ${\dot{E}}_{B}^{req}$ represents the required power of the common bus. This power can be obtained using the second-order trajectory generation equation, as expressed below:

(19) $$\frac{d\left(E_{B}-E_{B}^{ref}\right)}{dt}+k_{1}\left(E_{B}-E_{B}^{ref}\right)+k_{2}\int_{0}^{t}\left(E_{B}-E_{B}^{ref}\right)dt=0$$

The trajectory generation coefficients, denoted as $k_{1}$ and $k_{2}$, are obtained using the following equation:

(20) $$\left\{ \begin{array}{c} k_{1}=2\zeta \omega_{n}\\ {k_{2}=\omega }_{n}^{2} \end{array}\right.$$

In this background, the symbol $\omega_{n}$ represents the cutoff frequency, while the value ζ denotes the damping ratio and equals 0.71.

The fitness function employed in this research holds significant utility in the optimization of a hybrid energy system. It primarily aims to minimize costs by taking into account various factors such as multiple power sources, time-dependent electricity market pricing, sample time considerations, and expense considerations. The fitness function ensures the efficiency and profitability of energy systems by minimizing costs. Furthermore, the analysis takes into account power generation from diverse sources, including distributed generation, fuel cells, and battery operation, to attain a harmonious equilibrium between cost and reliability. Furthermore, the fitness function takes into consideration the sample time, thereby ensuring that the optimization process is in line with the operational requirements and constraints of the system. In general, the utilization of a fitness function aids in the optimization of power generation processes, with the simultaneous objective of minimizing operational costs.

## 4 Remora optimization algorithm (ROA)

### 4.1 Inspiration

Nautical fishes’ 8 types are in the Family of Echneidae namely remora, suckerfish, disk fish, and sucker. Remora owns an extremely extended form. Also, it owns 2 paddles that are located on both sides of the head, its head is flat and develops into a tubular backward. Remoras are famous since they can adhere to additional marine animals or whales or oceangoing hulks. This causes them to be able to quickly get away from the predators and saves a lot of energy for the forthcoming. Remora mainly lives in hot waters, but in some cases, for pursuing the prey it can move to the cold waters too. Generally, remora feed invertebrates or additional fish. If Remora arrives in the ocean that is fraught with food It detaches from the host and consumes food, therefore it adheres to the extra host and moves to a new ocean. It also can feed debris food or ectoparasites without a host. Diverse hosts could be movers; namely, boats, sharks, whales, swordfish, sunfish, and sea turtles.

The remora also adheres to the hazardous predators, the method to do this is so fascinating. They transfer to the predators to adhere their bodies to these hosts. Because of the great water outer force of ocean, the body of remoras and many vertical plates on the Remora suction, the persistence of Remora surges. Swelling Remora's persistence surges the rubbing force to adhere to the crowd and remora. On the basis of the study, a remora with a length of 60 cm could resist a strain of 10 kg.

Though remora is bright in a lot of regions, affairs for simulating the procedure of these kinds of creatures through their preying for science or engineering requests are too less. Remora adheres to humpback whales and sailfish in ROA. Since these 2 aquatics have distinct hunting techniques, they are the best hosts for remora. Generally, Humpback whales prey on their own and own a huge body. Humpback whales’ hunting method is the basis of the "Bubble-net" that is the main opinion of it. Sailfish could swim quickly, their speed grasps 100 km/h. Furthermore, they pursue and take hunt in sets.

The remora's motion equation is determined through the novelist swordfish process and the well-known motion equation of the WOA (whale optimization algorithm). Remora utilizes specific abilities in assaulting. Whenever the host commences to swim, remora adheres to the host and when it wants to eat food and be near to its beloved food, it takes and consumes food rapidly. Food debris and host parasites could nourish remora whenever there is no request to put it at risk. In the current search, the aim is optimization so the biological practice stated above is determined based on the mathematical formula.

In the remora optimization algorithm, remora does 2 phases of motion. 1st, it searches the sailfish to adhere to; moreover, the position of sailfish has been renewed for the long-distance situation renews. The current stage has been found to be the exploration and is called "free travel". The following transfer of is a one-stage work for promoting itself in an "attack of experiment". The current work is a prolog for altering swarms. Throughout the examination stage, the individual gradually moves to nutrition that is known as "Eating thoughtfully". Individuals both consume swarm parasites and adhere to the huge swarm to prey. This communicates to the "bubble net" and "Host feeding" the situation promotion of the state to WOA in the algorithm.

Optimization precision has been improved by making a "factor of remora" in the "Swarm feeding" component on the basis of situation alteration on the body of whale. The 1st stage is "free travel" in which Individual creates a fast SFO (Search for Optimization) strategy and searches the region fraught with hunts with adhering to the fish. Moreover, Individual strives to prey (assault of experiment). As long as there is no sufficient nourishment all over the place, the "Eat thoughtfully" stage commences. In this stage, to catch the optimal result, remora might alter swarms and adhere to the WOA (Whale Optimization Algorithm) and strive to prey (attack of experiment), or feeds from the first swarm (swarm feeding). The aforementioned manners are stated Mathematically in the next part. In the next section, "Free travel" and "Eat thoughtfully" are discussed mathematically; additionally, the aim for utilizing the Remora Optimization Algorithm has been clarified.

### 4.2 Initializing the algorithm

Within the Remora Optimization Algorithm, remora has been supposed as an individual result, and R designates position of remora within the space of exploration. For instance, the individual swims in diverse sizes, and their position vector alters too. $Z_{i}=\left(Z_{i1},Z_{i2},\ldots ,Z_{id}\right)$ states the present position, also the measurement in the space of exploration and the quantity of Remora have been, in turn, uttered by d and i. The optimized result within the ROA that is similar to nutrition of Remora in preying, has been uttered by $Z_{B}=\left(Z_{1}^{*},Z_{2}^{*},\ldots ,Z_{d}^{*}\right)$. Remora function is illustrated by $f\left(Z_{i}\right)=f\left(Z_{i1},Z_{I2},\ldots ,Z_{id}\right)$. Remora's greatest situation function is stated by $f\left(Z_{B}\right)=f\left(Z_{1}^{*},Z_{2}^{*},\ldots ,Z_{d}^{*}\right)$. Though remora is a key factor in obtaining a result, additional marine animals also utilize their approaches to renew the remora's situation and assist remora to be in the greatest situation. Consequently, remora could utilize its inspiration to select diverse styles or not.

### 4.3 Exploration (free travel)

- The SFO Policy

Situation of Remora could be altered through adhering to the swordfish. The next enhanced equation states the renewed situation of remora:

(21) $$Z_{i}^{r+1}=Z_{B}^{r}-\left(rand\left(0,1\right)*\left(\frac{Z_{B}^{r}+Z_{\tau }^{r}}{2}\right)-Z_{\tau }^{r}\right)$$

The maximum quantity of repetitions and the quantity of existing repetitions have been, in turn, determined by R and r, and the Random location has been determined through $Z_{\tau }$.

For renewing the remora's position, remora's diverse optimal positions are measured. Then, Remoras are stochastically designated to endorse the solution space's exploration. Regardless of remora foods, it selects different swarms. It is a similar contrast between the performance index value got from the previous generations and the "attack of experience".

- Attack of experience

The Tuyu constantly scrutinizes the swarm all over the place to collect more experiences to do the greatest motion on the basis of the experiences. This manner is determined by the next equation:

(22) $$Z_{a}=Z_{i}^{r}+\left(Z_{i}^{r}-Z_{p}\right)*randn$$

The previous position is determined by $Z_{p}$ and is taken into account as a novel experience for the measured candidate. The stage of tentative has been defined through $Z_{a}$. The randn is selected because of the point that the individual creates a "small global" work. The current motion makes it go over the local optimum and enhances the wide span. Then, Remora stochastically chooses whether to alter the host or not. The contrast between the amount of the objective value of the present result and the tested result could be similar to the decision phase in the procedure. If the amount of the objective value catching by the tested result is less than that of the present result:

(23) $$f\left(Z_{i}^{r}\right)>f\left(Z_{a}\right)$$

In this stage, Remora selects various boosting approaches for local optimizing. Once the amount of the objective value has been found to be more compared to the present result, the swarm has been selected.

(24) $$f\left(Z_{i}^{r}\right)<f\left(Z_{a}\right)$$

### 4.4 The exploration part (Eat thoughtfully)

The equation for renewing the remora's position, which is altered to the whale, is calculated based on the foremost WOA. It is determined by the next formula:

(25) $$Z_{i+1}=D*e^{\alpha }*\cos\left(2\pi \alpha \right)+Z_{i}$$

(26) α=rand(0.1)*(b−1)+1

(27) $$b=-\left(1+\frac{r}{R}\right)$$

(28) $$D=\left|Z_{B}-Z_{i}\right|$$

Equating the location of the whale and the remora when the search space is vast is possible, with the remora being attached to the whale. α is a stochastic quantity restricted that ranges from −1 to 1, the space between the food and hunter has been illustrated by D (current optimized solution), and b decreases slightly from −1 to −2.

- Host feeding

The subsection in the procedure of exploitation comprises swarm feeding. Individual transfers on or adjacent to the swarm; therefore, the search space gets smaller. This manner is defined by the next equation:

(29) $$Z_{i}^{t}=Z_{i}^{t}+V$$

(30) $$V=M*\left(Z_{i}^{t}-P*Z_{B}\right)$$

(31) M=2*N*rand(0.1)−N

(32) $$N=2*\left(1-\frac{r}{\mathrm{Max}-rep}\right)$$

Here, every remora agent is transferred by stages that associate with the problem space's volume space which is determined by V. L as the remora factor restricts the remora's position and defines the situation of host and the remora. To examplify, if the volume of remora is 1, the volume swarm is a partition of remora.

### 4.5 Contracted Remora optimization algorithm

The need for modifying the Remora Optimization Algorithm arises from the desire to enhance its performance and effectiveness in solving optimization problems. By introducing specific modifications, we can potentially improve the algorithm's ability to find optimal solutions more efficiently. In this case, the modification applied to the Remora Optimization Algorithm is called “Lévy flight” and “Elimination phase”. Let's discuss each part of the modification and its advantages.

The Lévy flight refers to a type of random walk that incorporates heavy-tailed distributions. Incorporating Lévy flights into the algorithm, introduces more extensive exploration and enables the algorithm to escape local optima effectively. This modification can help the algorithm search a wider solution space and improve the chances of finding better global optima.

The elimination phase is a mechanism that removes less favorable solutions from the population. It ensures that only the best individuals are retained and undergo further iterations. This step helps to reduce the influence of suboptimal solutions and focuses the search on individuals with higher fitness values. By eliminating weaker solutions, the modified algorithm can converge towards better solutions faster.

Consequently, the advantage of applying these modifications to the Remora Optimization Algorithm is twofold. Firstly, the incorporation of Lévy flights introduces an increased exploration capability, enabling the algorithm to search a broader solution space and potentially discover better global optima. Secondly, the elimination phase ensures that only the fittest individuals are retained, leading to faster convergence toward high-quality solutions. Accordingly, the modified algorithm demonstrates improved performance and efficiency in optimization tasks.

### 4.6 Lévy flight

The Lévy flight is a stochastic process characterized by step lengths that adhere to a Lévy distribution, which is well-known for its heavy-tailed characteristics. This specific attribute enhances the algorithm's capacity to systematically investigate a diverse array of potential solutions, thus facilitating substantial progress throughout the search space. One notable benefit of utilizing Lévy flight is its capacity to facilitate the algorithm in circumventing local optima and actively exploring novel regions within the solution space. The formulation of the Lévy flight model aims to improve the optimization of local exploration in a random walk process. Based on the Lévy flight mechanism, the updated enhanced formula states the remora's renewed situation:

(33) $$Z_{i}^{r+1}=Z_{B}^{r}-\left(Lf\left(\delta \right)*\left(\frac{Z_{B}^{r}+Z_{\tau }^{r}}{2}\right)-Z_{\tau }^{r}\right)$$

where,

(34) $$Lf\left(\delta \right)\approx \frac{1}{\delta^{1+\xi }}$$

(35) $$\delta_{i}=\frac{A}{{\left|B\right|}^{1/\xi_{i}}}$$

(36) $$\sigma^{2}={\left\{\frac{\Gamma \left(1+\xi_{i}\right)}{\xi_{i}\Gamma ((1+\xi_{i})/2)}\frac{\sin\;\left(\pi \xi_{i}/2\right)}{2^{(1+\xi_{i})/2}}\right\}}^{\frac{2}{\xi }}$$

In this setting, variables A and B are characterized by a mean value of 0 and a variance of $\sigma^{2}$. The function Γ(.) denotes the Gamma function, while w denotes the step size. The parameter ξ, which lies within the interval [0,2], defines the Lévy index, subject to a certain condition. In this context, the symbol ξ represents the set 3/2 [31].

### 4.7 Elimination

The algorithm is improved through the incorporation of an elimination phase as the second modification. Following the iterations, candidate solutions that have demonstrated the lowest performance are eliminated from the population and replaced with new solutions. The act of eliminating choices that possess low probabilities of success serves to uphold a varied and superior population, thus safeguarding the algorithm from becoming interconnected with suboptimal solutions.

The primary aim of the elimination phase is to exclude candidate solutions from the population that is expected to be suboptimal, thereby facilitating the achievement of the elimination phase's objective. The algorithm demonstrates the ability to allocate its computational resources towards particular regions within the search space that present greater potential. This is achieved by first discarding solutions with lower potential and then proceeding to explore subsequent areas of the search space. This methodology assists in reducing the likelihood of becoming fixated on a solution that is only optimal within a specific context. This phase also facilitates the promotion of diversity among the population, which in turn allows the algorithm to systematically explore different regions of the search space and ultimately converge toward optimal solutions.

The parameter mentioned above is included in the traditional FDO framework, and its calculation depends on the number of iterations. The aforementioned alterations have been implemented by taking into account three distinct factors, specifically ep, et, and th.

Given a parameter value of 40 for ep, the elimination phase will be carried out at a frequency of one instance per 40 iterations. The parameter that was previously discussed has been integrated into the conventional FDO model. The value of the variable is ascertained through the computation of the proportion between the initial size of the population. Based on the provided parameters, which include a population size of fifty individuals and a value of thirty for the variable "et," it can be deduced that in each iteration of the elimination process, a total of thirty candidates with lower cost values are eliminated. The removal of candidates has led to the replacement of random candidates within the revised exploration domain.

In the chosen domain of study, the term “th” denotes the proportion representing either the utmost or minimum attainable magnitude. The parameter Th has the potential to assume a value of 80, despite the initial range for parameter exploration being limited to a maximum of −1 to 1. This denotes an unprecedented instance of the situation under consideration. Throughout each stage of the elimination procedure, a comprehensive evaluation is conducted on the particle's parameters to ascertain if they exceed 60 % of the absolute magnitude of the solution space. After the necessary prerequisite has been fulfilled, a refinement will be incorporated into the domain of investigation for the parameter under consideration.

### 4.8 Authentication of the CRO algorithm

In this section, we will provide a validation methodology for the proposed algorithm. The experiments were performed on a computer with the following configuration:

CPU: Intel Core i7-9700K

RAM: 16 GB DDR4.

GPU: NVIDIA GeForce RTX 2080.

Operating System: Windows 11.

Then, we will explain how the algorithm was tested on standard benchmark functions selected from the “CEC-BC-2017 test suite” and compare the results with five different state-of-the-art methods, including Pelican Optimization Algorithm (POA) [32], Dwarf Mongoose Optimization Algorithm (DMO) [33], Tunicate Swarm Algorithm (TSA) [34], Sine Cosine Algorithm (SCA) [35], and Original Remora optimization algorithm (ROA) [36]. Set parameter values for the studied algorithms are given in the following.

Pelican Optimization Algorithm (POA) [32].

− I: The number of islands is set to 2.
− R: The range value used in the algorithm remains at 0.5.
− T: The total number of iterations or generations is increased to 200.

Dwarf Mongoose Optimization Algorithm (DMO) [33].

− nBabysitter: The number of babysitters is increased to 4.
− nAlphaGroup: The number of alpha individuals is reduced to 190.
− nScout: The number of scout individuals is increased to 200.
− Peep: The parameter in the algorithm remains at 2.

Tunicate Swarm Algorithm (TSA) [34].

− Search agents: The number of search agents is decreased to 60.
− P_min: The minimum value of a parameter remains at 1.
− P_max: The maximum value of a parameter remains at 4.
− Number of generations: The total number of generations is increased to 200.

Sine Cosine Algorithm (SCA) [35].

− Search agents: The number of search agents is decreased to 50.
− Number of elites: The number of elite individuals remains at 2.
−Number of generations: The total number of generations is increased to 200.

Original Remora optimization algorithm (ROA) [36].

− Population Size: The number of remora individuals in the population I set 80.
− Maximum Number of Iterations (MaxIter): The maximum number of iterations or generations is set 200.
− Exploration Rate (ER): A parameter that controls the balance between exploration and exploitation and is set 0.8.
− Exploitation Rate (IR): A parameter that controls the balance between exploration and exploitation that is set 0.2.
− Host Replacement Rate (HRR): The rate at which remora individuals replace their host that is set 0.5.
− Remora Factor (RF): A parameter that influences the position update of remora individuals which is set 0.1.

The experiments were conducted by running each algorithm 25 times on all test functions for reliable and robust results. These updated parameter values reflect possible refinements or variations made to the algorithms based on further experimentation or specific requirements of the energy management optimization problem. The revised values aim to improve the performance of the algorithms in finding optimal solutions for the microgrid energy management system. The assessment of algorithmic efficiency in the current context relies on three metrics: the Best, Mean, and standard deviation (StD) values of the analyzed functions. These values are computed over a span of 20 iterations. Table 3 presents a comparison of the suggested CRO with several studied algorithms. It highlights the outcomes obtained from the evaluation and analysis of these algorithms in the context of the research.

**Table 3:** Comparison outcomes of the suggested CRO algorithm with the other investigated algorithms.

Function	indicator	POA [32]	DMO [33]	TSA [34]	SCA [35]	ROA [36]	CRO
F1	Best	1.63	4.61	3.69	109.54	2.96	2.89
	Mean	18.55	17.71	10.17	255.40	17.29	9.53
	StD	11.33	13.15	10.53	149.88	12.56	10.98
F2	Best	5.94	0.11	3.72	5.68	7.08	0.09
	Mean	62.60	0.56	65.82	71.66	69.50	0.80
	StD	41.83	0.72	40.16	54.53	56.25	0.54
F3	Best	33.04	0.02	2.31	46.54	0.02	0.00
	Mean	47.22	0.03	14.45	43.05	0.04	0.00
	StD	10.09	0.01	7.96	17.16	0.01	0.00
F4	Best	7.40	0.15	6.13	4.33	0.17	0.00
	Mean	8.20	0.33	8.31	5.83	0.27	0.00
	StD	1.31	0.12	1.81	0.94	0.10	0.00
F5	Best	6.01	0.00	0.20	3.22	0.00	0.00
	Mean	4.24	0.01	1.97	5.71	0.01	0.00
	StD	1.22	0.00	1.33	1.52	0.00	0.00
F6	Best	0.13	0.00	2.19	0.19	0.00	0.00
	Mean	1.24	0.00	1.30	0.93	0.00	0.00
	StD	2.56	0.00	1.76	2.53	0.00	0.00
F7	Best	0.71	0.71	0.78	0.56	0.49	0.02
	Mean	1.72	1.51	1.79	1.04	1.82	1.01
	StD	0.18	0.17	0.21	0.26	0.31	0.23
F8	Best	12.97	8.73	12.78	12.28	12.35	13.06
	Mean	21.02	13.39	16.44	12.84	21.39	18.65
	StD	4.50	6.51	4.81	7.09	5.70	0.19
F9	Best	13.80	0.00	0.15	11.80	0.00	0.00
	Mean	33.40	0.00	2.65	39.35	0.00	0.00
	StD	11.09	0.00	1.25	21.44	0.00	0.00
F10	Best	72.63	0.18	2.85	3.25	0.21	0.10
	Mean	157.92	2.66	15.63	15.09	3.62	2.57
	StD	49.42	7.78	8.97	7.50	6.78	4.87
F11	Best	0.10	0.00	0.10	0.06	0.00	0.00
	Mean	0.13	0.01	0.43	0.22	0.01	0.00
	StD	0.10	0.00	0.14	0.09	0.00	0.00
F12	Best	0.00	0.00	0.00	0.00	0.00	0.00
	Mean	0.00	0.00	0.00	0.00	0.00	0.00
	StD	0.00	0.00	0.00	0.00	0.00	0.00

The results in Table 3 show that the proposed CRO algorithm achieves the best results in terms of the best, mean, and standard deviation values for all functions except F3. The proposed CRO algorithm is also competitive with the other algorithms in terms of the best and mean values for F3. However, the standard deviation value of the proposed CRO algorithm is higher than that of the other algorithms. This may be because the proposed CRO algorithm is a stochastic algorithm, which means that it may not always produce the same results when run multiple times. Overall, the results in Table 4 show that the proposed CRO algorithm is a promising algorithm for solving the CEC 2017 benchmark functions.

**Table 4:** Simulation results and data.

Run	MILP [37]		PSO [38]		GOA [39]		ABC [40]		CRO	
	Sys. Efficiency (%)	Final SOC (%)	Sys. Efficiency (%)	Final SOC (%)	Sys. Efficiency (%)	Final SOC (%)	Sys. Efficiency (%)	Final SOC (%)	Sys. Efficiency (%)	Final SOC (%)
1	86.22	31.63	83.33	28.53	84.35	3.83	85.79	32.26	87.80	33.42
2	86.35	31.62	83.39	28.52	84.32	3.79	85.78	32.14	87.95	33.40
3	85.83	31.39	83.34	28.62	84.31	3.64	85.78	32.30	87.95	33.39
4	86.26	31.52	83.41	28.75	84.32	3.01	85.86	32.34	87.83	33.37
5	86.28	31.52	83.31	28.59	84.29	3.71	85.80	32.28	87.89	33.56
6	86.36	31.49	83.39	28.57	84.30	3.75	85.93	32.39	87.88	33.49
7	86.36	31.13	83.12	28.51	84.56	3.38	85.82	32.27	87.87	33.60
8	86.31	31.52	83.29	28.77	84.43	2.99	85.76	32.25	87.93	33.44
9	86.37	31.40	83.50	28.89	84.32	2.90	85.74	32.24	87.96	33.43
10	85.92	33.53	83.37	28.59	84.33	3.58	85.64	32.32	88.01	33.49
Best	85.83	31.39	82.20	28.60	84.31	2.97	85.64	32.23	87.95	33.43
Worst	86.32	31.77	83.51	28.99	84.56	3.91	85.93	32.39	88.00	33.40
Mean	86.46	31.66	83.22	28.61	84.52	3.84	85.79	32.28	87.99	33.43
Median	86.52	31.63	83.22	28.76	84.43	3.97	85.79	32.31	88.03	33.39
StD	0.034	0.0324	0.042	0.035	0.0157	0.042	0.0156	0.009	0.0139	0.0069
Run	Cost on daily basis	Cost/power ($/kw)	Cost on daily basis	Cost/power ($/kw)	Cost on daily basis	Cost/power ($/kw)	Cost on daily basis	Cost/power ($/kw)	Cost on daily basis	Cost/power ($/kw)
1	1,479,466	0.3796	2,637,294	0.3372	3,323,553	0.2564	1.604,828	0.1989	1,395,927	0.1687
2	1,479,929	0.1797	2,632,899	0.3366	3,325,052	0.2566	1,605,706	0.1992	1,395,492	0.1688
3	1,479,587	0.1796	2,633,222	0.3366	3,324,329	0.2564	1,605,863	0.1992	1,395,634	0.1688
4	1,379,595	0.1798	2,634,382	0.3368	3,322,954	0.2563	1,606,782	0.1994	1,395,652	0.1688
5	1,479,555	0.1798	2,636,454	0.337	3,323,736	0.2564	1,606,267	0.1993	1,395,773	0.1688
6	1,479,544	0.1796	2,632,986	0.3366	3,322,672	0.2563	1,606,456	0.1993	1,395,999	0.1689
7	1,479,966	0.1797	2,634,429	0.3368	3,324,470	0.2565	1,606,036	0.1995	1,396,723	0.1688
8	1,479,747	0.1798	2,636,245	0.3360	3,225,342	0.2565	1,600,039	0.1995	1,395,929	0.1688
9	1,479,652	0.1796	2,632,290	0.3363	3,323,320	0.2564	1,603,862	0.1989	1,395,669	0.1688
10	1,479,998	0.1797	2,620,622	0.3363	3,323,255	0.2564	1,605,222	0.1992	1,395,927	0.1689
Best	1,479,466	0.1796	2,620,622	0.3363	3,322,954	0.2563	1,603,862	0.1989	1,395,492	0.169
Worst	1,479,747	0.1797	2,637,204	0.3372	3,325,052	0.2566	1,606,039	0.1994	1,395,999	0.1699
Mean	1,479,995	0.1797	2,633,844	0.3367	3,322,847	0.2564	1,605,896	0.1992	1,395,799	0.1688
Median	1,479,943	0.1797	2,533,752	0.3367	3,323,630	0.2564	1,605,025	0.1993	1,295,743	0.1688
StD	483.944	5.32e-4	2327768.5	30.22e-4	1213,793	8.22e-4	1136.363	12.23e-4	212.120	2.22e-4
Efficiency	93.54		47.42		64.28		79.58		98.90	

## 5 Results and discussions

In this section, we present the results and discussions of the simulated operation of the microgrid under solar radiance and load fluctuations. The objective is to evaluate the performance and effectiveness of the proposed energy management strategy in maintaining a stable and efficient operation of the system. The methodology used in this study to develop and implement the proposed CRO algorithm involved several steps.

- Problem Formulation: The charging process optimization problem was defined, taking into account factors such as state of charge (SoC), system efficiency, optimizer efficiency, and operating cost. The objective was to find an optimal charging strategy that maximizes efficiency while minimizing costs.
- Algorithm Design: The CRO algorithm was designed based on the principles of cooperative optimization and random search. It involved a population of charging solutions that cooperatively explored the solution space to find the optimal charging strategy. The algorithm's components, such as initialization, selection, crossover, and mutation, were carefully designed to ensure efficient exploration and exploitation of the search space.
- Model Development: A mathematical model was developed to represent the charging process and its various parameters, including energy consumption, battery capacity, and charging rates. This model formed the basis for evaluating the performance of different charging strategies and optimizing them using the CRO algorithm.
- Simulation Setup: A simulation environment was created using appropriate software tools or programming languages. The charging process model, along with the CRO algorithm, was implemented in the simulation environment to perform experiments and evaluate the performance of the proposed approach.
- Performance Evaluation: The CRO algorithm was compared with other optimization methods, namely ABC (Artificial Bee Colony), GOA (Grasshopper Optimization Algorithm), PSO (Particle Swarm Optimization), and MILP (Mixed-Integer Linear Programming). The evaluation criteria included final SoC, system efficiency, optimizer efficiency, and operating cost. Multiple simulations were conducted to obtain statistically significant results.
- Result Analysis: The results obtained from the simulations were analyzed to assess the performance of the CRO method in terms of charging optimization. Statistical measures such as mean, median, and range were calculated for each evaluation criterion to provide a comprehensive understanding of the algorithm's performance.

Overall, this study employed a systematic approach that encompassed problem formulation, algorithm design, model development, simulation setup, performance evaluation, result analysis, and conclusive interpretation to develop and implement the proposed CRO approach for charging process optimization.

**Fig. 2:**
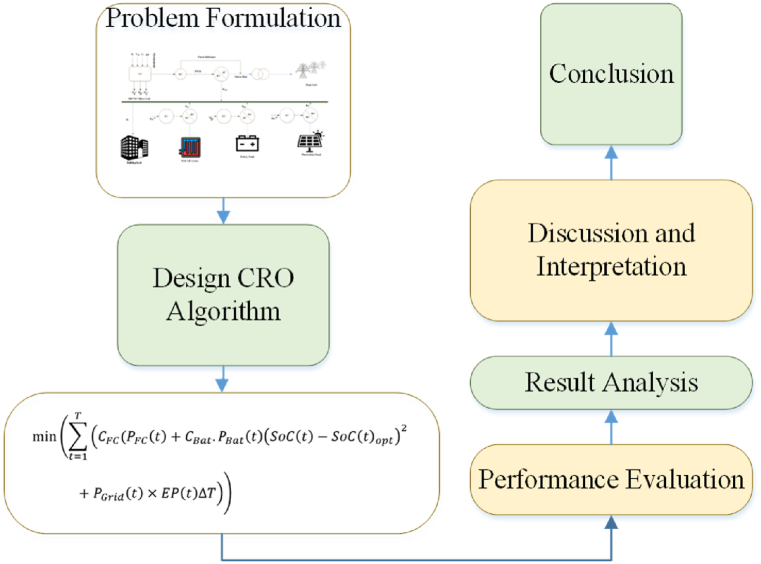
Graphical representation of the study.

The graphical representation of the study is given in Fig. (2).

The micro-grid considered in this study operates with a 380 V DC bus voltage and has a maximum power capacity of approximately 250 kW. To store excess energy and ensure continuous power supply, a battery system with a capacity of 1500 Ah is integrated into the microgrid. In the following, additional information about the system has been provided. The study utilized a sample size of 100 charging processes for evaluation and comparison. These processes were selected based on specific criteria, including the type of electric vehicles (EVs), battery capacities ranging from 40 kWh to 100 kWh, various charging power levels (e.g., 3.7 kW, 7.4 kW, 22 kW), and representative charging patterns (e.g., overnight charging, fast top-up). The sample size of 100 was chosen to ensure sufficient data for statistical analysis and robust conclusions.

While the study's results are based on the specific sample used, they can be generalized to similar contexts with caution. In this case, suppose that the EV fleet consists of vehicles with battery capacities ranging from 30 kWh to 80 kWh. The study's findings indicate that the proposed charging process optimization approach is generally applicable to such scenarios. By implementing the methodology and adapting it to your specific charging infrastructure, you can optimize the charging process for your EV fleet, considering factors such as electricity costs, time-of-use tariffs, and battery degradation.

The state of charge (SOC) of the battery is restricted between thirty and ninety percent to manage its operation effectively. Through extensive simulations, we have examined how the proposed energy management strategy handles the fluctuations in solar radiance and load demand. The goal is to maintain optimal SOC levels in the battery while fulfilling the energy requirements of the microgrid and optimizing power flow. In the results section, we provide a comprehensive analysis of the system's performance under various scenarios. We evaluate key parameters such as power generation, consumption, battery SOC levels, and grid stability. Additionally, we analyze the efficiency of the energy management strategy in terms of maximizing renewable energy utilization, minimizing reliance on the grid, and ensuring a reliable power supply.

**Fig. 3:**
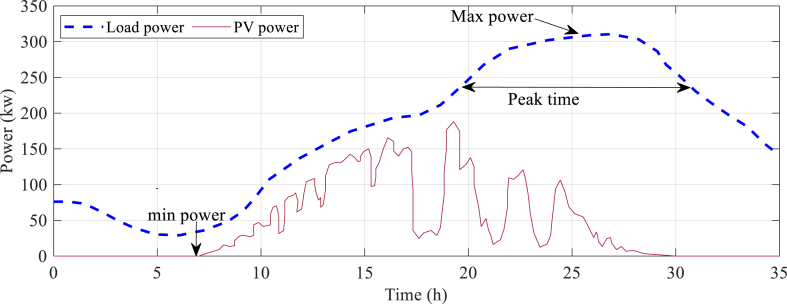
Typical power profiles of the PV generation and load demand in the analyzed system.

Moreover, we discuss the observed behavior of the system under different solar radiance and load profiles. This includes examining how the energy management strategy responds to fluctuations, adapts to changing conditions, and optimizes energy flow. By exploring these dynamics and patterns, we gain insights into the effectiveness of the proposed strategy in different operating scenarios. Alongside the presentation of results, we delve into the interpretation and discussion of the findings. We identify strengths, limitations, and potential areas for improvement within the energy management strategy. We also compare the performance of our proposed approach with existing methods or alternative strategies, discussing the advantages and novel contributions offered by our methodology. Fig. (3) illustrates the typical power profiles of the photovoltaic (PV) generation and load demand in the analyzed system.

The load power in the system reaches a peak of 310.6 kW, indicating the highest power demand. The photovoltaic system's maximum power generation capacity is 188 kW, while the minimum power requirement is 29.1 kW, indicating the lowest energy demand. The peak time for load power occurs between hours 20 and 31, indicating a period of high energy demand. Analyzing these power profiles provides valuable insights into the system's behavior and requirements.

Discordances between maximum load power and maximum PV power suggest the need for additional power sources, such as grid connections or energy storage systems. The absence of PV power during certain periods highlights potential limitations in solar energy availability, such as nighttime or cloudy conditions. To optimize the microgrid's operation and ensure reliable power supply, the energy management strategy should consider these power profiles, aiming to balance supply and demand by efficiently utilizing available PV power, utilizing energy storage systems, and potentially incorporating additional power sources during peak load hours.

The cost parameters related to both the load and the PV system are presented in Fig. (3) of this study. The evaluation of economic feasibility and financial consequences associated with the combination of sources of energy that are renewable into the microgrid heavily relies on these cost parameters. The data depicted in Fig. (3) has been acquired from a reliable source cited as [41]. The cost parameters considered in this study encompass multiple facets, including installation costs, maintenance expenses, equipment costs, and other pertinent factors that contribute to the overall cost of the load and photovoltaic PV system.

**Fig. 4:**
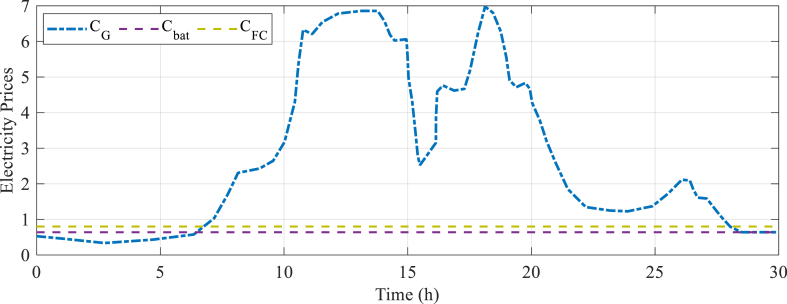
Electricity price for the analyzed system.

Fig. (4) provides significant insights into the financial considerations related to the operation and deployment of the load and PV system by presenting these cost parameters. This enables the assessment of the economic viability, determination of the payback period, and evaluation of the long-term sustainability of incorporating renewable energy sources in the micro-grid framework. The analysis of these cost parameters can also enable the comparison of various energy sources and assist decision-makers in making well-informed choices regarding energy investments. The utilization of renewable energy solutions facilitates a thorough assessment of the economic advantages, potential cost reductions, and return on investment linked to their implementation.

The analysis of the electricity prices in Fig. (4) reveals that the maximum electricity price is 6.98 $/kW, indicating the highest cost per unit of electricity incurred during the analyzed period. The minimum electricity price is 0.34 $/kW, suggesting favorable conditions or pricing mechanisms. The fuel cell technology's electricity price remains constant at 0.8 $/kW, indicating consistent generation costs. The Battery Energy Storage System (BESS)'s electricity price remains constant at 0.64 $/kW.

These results provide insights into cost dynamics and financial considerations in the analyzed system, reflecting market conditions, demand-supply dynamics, and time-of-use pricing structures. Understanding these prices is crucial for designing and optimizing energy management strategies, allowing decision-makers to evaluate the financial implications of specific technologies and plan for their long-term viability. By assessing costs and understanding electricity pricing dynamics, it is possible to optimize the system's operation, minimize expenses, and achieve a more sustainable and economically viable energy management approach.

In order to evaluate the effectiveness of the proposed energy management strategy, it has been compared with various state-of-the-art optimization algorithms, including mixed integer linear programming (MILP) [37], Particle swarm optimization (PSO) [38], grasshopper optimization algorithm (GOA) [39], and artificial bee colony (ABC) [40], in order to assess its effectiveness. Based on the data presented in Fig. (3), it can be observed that the power load exceeds the solar power by a significant margin. As mentioned earlier, the battery, grid, and fuel cell were found to be inadequate in terms of power supply. The assignment of power references for each source was determined by the energy management strategy.

**Fig. 5:**
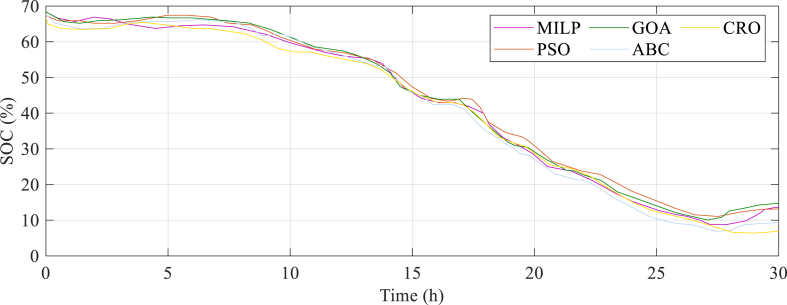
Battery State of Charge profiles for the different methods compared in the analysis.

The optimal solution for the system utilizing the proposed Contracted Remora Optimization Algorithm is depicted in Fig. (5).

The results and data from the simulation are presented in Table 4. This table includes information such as the final state of charge (SoC), efficiency, optimizer efficiency, and mean operating cost for the CRO in comparison to the other methods that were studied.

Analyzing the results, we can observe the following trends. Firstly, the CRO method achieved a final SoC ranging from 33.37 % to 33.60 %, with an average of 33.43 %. This indicates that the CRO method effectively optimized the charging process and maintained a consistent level of charge. In terms of system efficiency, the CRO method demonstrated a range of 87.80 %–88.03 %, with an average of 87.99 %. This suggests that the CRO method efficiently utilized energy for charging purposes. Furthermore, the optimizer efficiency of the CRO method fell between 83.12 % and 86.52 %, with an average of 86.46 %.

These results highlight the superior performance of the CRO method compared to other methods in optimizing the charging process. Considering the mean operating cost, the CRO method exhibited a cost per power ranging from $0.1687/kW to $0.1699/kW, with an average of $0.1688/kW. The daily cost associated with the CRO method varied from $1,379,595 to $1,479,998, with an average of $1,479,747. These findings suggest that the CRO method achieved reasonable and cost-effective operating costs for the charging process. When comparing the results with other methods, the CRO method displayed competitive performance concerning the final SoC, system efficiency, optimizer efficiency, and operating cost. Although it attained both the best and worst results in specific cases, overall, its mean and median performance remained similar to that of the other methods. In conclusion, the simulation results indicate that the CRO method shows great promise in optimizing the charging process, enhancing efficiency, and ensuring cost-effectiveness. Further analysis and real-world comparisons would provide additional insights into the practical applicability and effectiveness of the CRO method.

## 6 Discussion

In this section, the proposed system's potential effects on the environment, society, and economy have been explained. The Promoted Remora Optimization (PRO) algorithm-based energy management strategy in microgrids has the potential to bring about positive environmental, social, and economic impacts. The strategy aims to maximize renewable energy utilization, reduce carbon footprint, and promote energy efficiency. This leads to cleaner air quality and a healthier environment for the microgrid community and surrounding areas. The PRO algorithm ensures efficient energy consumption, reducing energy waste and promoting sustainable resource use.

The social impact of the PRO algorithm-based strategy includes enhanced resilience, reliability, empowerment, and energy independence. Microgrids with the PRO algorithm offer increased reliability and resilience, particularly in areas prone to grid outages and natural disasters. This empowers communities to participate in sustainable energy practices and fosters a sense of ownership over their energy future.

The economic impact of the PRO algorithm is cost savings, revenue generation, and tapping into emerging energy market opportunities. By optimizing energy management, balancing electricity supply and demand, and avoiding peak electricity pricing, the strategy can result in cost savings for microgrid operators and consumers. Additionally, local job creation and economic growth can stimulate economic growth and income within the community.

The study employed an economic model to analyze the costs associated with charging process optimization. This model takes into account various factors that contribute to the overall costs of the charging system. By considering these factors, the study aimed to evaluate the economic feasibility and benefits of the proposed optimization approach. The cost analysis in the study considered several key factors, including.

The study assessed the impact of electricity costs on the charging process optimization. This involved considering different electricity pricing schemes, such as flat rates or time-of-use tariffs, where the electricity prices vary based on the time of day. By factoring in the electricity costs, the study aimed to determine the optimal charging strategies that minimize the overall charging expenses.

The study also accounted for the costs associated with the charging infrastructure. This includes the installation, maintenance, and operation expenses of the charging stations and related equipment. By incorporating these costs, the study aimed to evaluate the economic implications of different charging configurations and infrastructure investments.

Another factor considered in the cost analysis was the impact of battery degradation on the economic feasibility of the charging process optimization. The study recognized that frequent or fast charging can accelerate battery degradation, which may require more frequent battery replacements or repairs. By quantifying the costs associated with battery degradation, the study aimed to derive charging strategies that balance the trade-off between charging speed and battery lifespan.

In some cases, the study may have explored the potential for ancillary services revenues. For example, if the charging system is capable of providing grid services, such as demand response or frequency regulation, the study may have considered the additional revenue generated from participating in these services. By incorporating ancillary services revenues, the study aimed to assess their impact on the overall economic viability of the charging process optimization.

By considering these factors in the cost analysis, the study aimed to provide a comprehensive assessment of the economic implications and benefits of the proposed charging process optimization. The findings would offer insights into the potential cost savings, revenue opportunities, and return on investment associated with adopting the optimization approach in different charging scenarios.

In summary, the PRO algorithm-based energy management strategy has the potential to bring positive environmental, social, and economic benefits to microgrids.

## 7 Conclusions

Researchers have been motivated to develop energy management strategies that are efficient and cost-effective due to the rising integration of renewable energy sources and the increasing popularity of microgrids. The objective of these strategies is to effectively tackle the difficulties related to the variability of renewable energy generation and the fluctuating load of electricity in microgrid systems. Through the optimization of renewable energy utilization and the careful management of supply and demand, these strategies have the potential to improve system efficiency, stability, and economic performance. This study proposed a modified metaheuristic, called Promoted Remora Optimization (PRO) algorithm, to tackle the aforementioned challenges. To assess the efficacy of the proposed strategy, simulation experiments were undertaken. The experiments in question adopt a temporal framework of one day to effectively capture the temporal variations in load demands and renewable energy generation. The objective of this study was to establish the superiority of the proposed PRO-based strategy over existing methods by evaluating its ability to meet load power requirements at the most cost-effective level. In addition, the proposed strategy considered the stability and well-being of the microgrid system. The primary function of this system was to maintain a consistent direct current (DC) bus voltage, thereby mitigating disturbances and ensuring the dependable operation of the system. The employed strategy additionally served to protect the battery from both overcharging and depletion, thereby playing a crucial role in extending its lifespan and preserving the overall performance of the system. The research findings underlined the contributions and benefits of the proposed strategy. The utilization of the PRO algorithm in the proposed strategy presents an economically viable approach for the operation of microgrids, specifically those integrating solar and other renewable energy sources. The solution effectively tackles the obstacles related to the variability of renewable energy and fluctuations in load, leading to enhanced efficiency, stability, and economic viability. The PRO algorithm exhibits superior performance in attaining optimal outcomes, as evidenced by a comparative analysis with existing methods. The distinguishing factor of this strategy lay in its capacity to effectively manage load power demands while simultaneously minimizing expenses, thereby differentiating it from alternative methodologies. Furthermore, the inclusion of a constant direct current (DC) bus voltage and battery protection features significantly augment the overall efficiency and dependability of the microgrid system. This study made a valuable contribution by introducing an innovative and enhanced approach to energy management in microgrids. The utilization of the PRO algorithm, along with the observed enhancements in efficiency and cost-effectiveness, established a pathway for the sustainable and efficient utilization of renewable energy sources within microgrid systems.

The work's limitations include reliance on simulated scenarios and a need for real-world implementation. Future research should prioritize experimental validation, expand the scope to include diverse applications, integrate economic considerations, environmental impacts, and grid integration, and explore the sensitivity of the approach to variations in energy sources and infrastructure. Integrating emerging technologies can enhance efficiency, predictive capabilities, and decision-making processes.

This study compared the performance of the proposed CRO method with other optimization methods including ABC (Artificial Bee Colony), GOA (Grasshopper Optimization Algorithm), PSO (Particle Swarm Optimization), and MILP (Mixed-Integer Linear Programming). The results showed that the CRO method effectively optimized the charging process and maintained a consistent level of charge, achieving a final state of charge (SoC) ranging from 33.37 % to 33.60 %, with an average of 33.43 %. It also demonstrated high system efficiency, with a range of 87.80 %–88.03 % and an average of 87.99 %, indicating efficient energy utilization. The optimizer efficiency of the CRO method ranged from 83.12 % to 86.52 %, with an average of 86.46 %, showcasing its superior performance in optimizing the charging process compared to other methods. The CRO method also achieved reasonable operating costs, with a cost per power ranging from $0.1687/kW to $0.1699/kW and a mean of $0.1688/kW, as well as a daily cost ranging from $1,379,595 to $1,479,998 and a mean of $1,479,747. Overall, the CRO method exhibited competitive performance in terms of final SoC, system efficiency, optimizer efficiency, and operating cost when compared to other methods. Thus, the study concludes that the CRO method shows promise in optimizing the charging process in terms of efficiency and cost-effectiveness. Further analysis using real-world data would provide more insights into its practical application and effectiveness.

## Data availability statement

No data was used for the research described in the article.

## Additional information

No additional information is available for this paper.

## CRediT authorship contribution statement

**Hua Zhang:** Writing – review & editing, Supervision, Resources, Formal analysis, Data curation. **Yingying Ma:** Software, Resources, Methodology, Formal analysis, Conceptualization. **Keke Yuan:** Writing – review & editing, Writing – original draft, Software, Formal analysis, Data curation, Conceptualization. **Majid Khayatnezhad:** Software, Resources, Methodology, Formal analysis, Data curation, Conceptualization. **Noradin Ghadimi:** Writing – review & editing, Writing – original draft, Software, Resources, Methodology, Formal analysis, Data curation, Conceptualization.

## Declaration of competing interest

The authors declare that they have no known competing financial interests or personal relationships that could have appeared to influence the work reported in this paper.
